# Conductivity Enhancement and Strain Gauge Optimization for Accurate and Stable Flexible Wearable Motion Sensors

**DOI:** 10.1021/acsomega.5c10697

**Published:** 2026-01-14

**Authors:** Yen-Kai Huang, Shih-Chen Shi, Dieter Rahmadiawan, Guan-Yu Chen

**Affiliations:** † Department of Mechanical Engineering, 34912 National Cheng Kung University, Tainan 70101, Taiwan; ‡ Department of Mechanical Engineering, Universitas Negeri Padang, Padang, Sumatera Barat 25173, Indonesia

## Abstract

Flexible wearable sensors are widely used in health monitoring and sports training, but their accuracy and stability are often limited by poor conductivity and unsuitable strain gauge design. Conductive hydrogels offer softness and biocompatibility, yet PEDOT:PSS-based systems typically suffer from unstable signals and limited long-term reliability. Enhancing conductivity and aligning the strain gauge orientation with muscle motion are crucial to improving performance. This study developed a dual-network poly­(vinyl alcohol) (PVA)/poly­(acrylic acid) (PAA) hydrogel containing dimethyl sulfoxide (DMSO)-modified poly­(3,4-ethylenedioxythiophene):poly­(styrenesulfonate) (PEDOT:PSS) for wearable motion sensing. DMSO was introduced as a secondary dopant, and FTIR, resistivity measurements, and DMA evaluated its effect. Two sensor designs, a biaxial strain gauge and a strain rosette, were fabricated using laser cutting and casting, and applied to shoulder joint monitoring during bench press and shoulder press training. Long-term stability was assessed using dynamic error control charts over 21 days. The results showed that DMSO reduced resistivity by up to 80% at 15 wt % and improved stability at 5 wt %. Motion tests confirmed that the biaxial gauge outperformed the rosette in multiplane monitoring with mean errors below 6°. Stability analysis further revealed that 5 wt % DMSO samples maintained reproducibility for 21 days, while untreated samples degraded after 14 days. These findings confirm that DMSO-enhanced hydrogels and biaxial strain gauge design provide accurate, stable, and practical wearable sensors.

## Graphical abstract

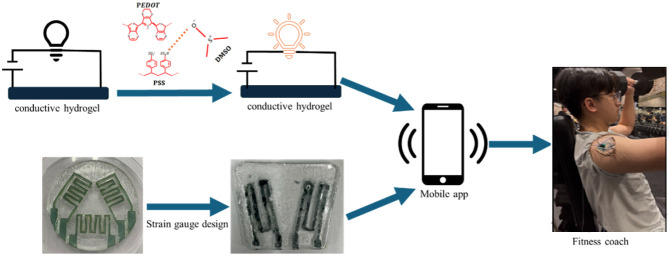

## 1 Introduction

Wearable devices are flexible electronics that attach to the skin and are increasingly applied in health monitoring, sports tracking, and intelligent interconnection. The global market for flexible electronics is projected to grow from 22 billion USD in 2022 to 89.2 billion USD by 2030, with wearable sensors as the fastest-growing segment. In 2020, their market value reached 1.5 billion USD, with a compound annual growth rate of 10.7%.^1,2^ Conductive hydrogels have attracted attention because they combine softness, stretchability, and skin compatibility, making them ideal for wearable sensing.^3^ Poly­(vinyl alcohol) (PVA) has provided elasticity and biocompatibility as a hydrogel matrix,^4,5^ while poly­(styrenesulfonate) (PEDOT:PSS) has demonstrated excellent conductivity and stability for sensing materials.^6^ However, limitations remain in conductivity, strain gauge design, and system accuracy, underscoring the need for innovation.

Researchers have enhanced hydrogel conductivity by adding metallic nanoparticles, carbon materials, or conductive polymers.^7–15^ Inorganic fillers achieve high conductivity but lack flexibility, leading to signal distortion during motion. Conductive polymers and ionic materials better balance conductivity with softness. Organic solvents such as ethylene glycol (EG), polyethylene glycol (PEG), and dimethyl sulfoxide (DMSO) have improved PEDOT:PSS.^16–18^ The sulfonic acid groups of PSS stabilize PEDOT but act as insulating layers that increase resistance.^19^ DMSO doping has replaced PEDOT:PSS interactions with hydrogen bonding, removed excess PSS, and improved conductivity.^20^

Strain gauges are the core of motion measurement and include uniaxial, planar multiaxial, and three-dimensional types.^21–24^ Misaligned placement can produce nonlinear deformation and reduce accuracy, since the principal strain *ε_x_* may be influenced by both *ε_x_* and shear strain *γ_xy_*. Customized gauge arrays have been designed to match motion modes and enhance signal reliability.^25^ Recent developments emphasize that optimizing the geometry and placement of hydrogel strain sensors is as crucial as improving material conductivity. Novel designs such as biaxial or multiaxial topologies have demonstrated improved sensitivity and directional accuracy during complex joint movements.^26^

Finally, accuracy and stability are essential performance indicators. Resistive sensors generate resistance changes under deformation and show potential.^27,28^ Stability has been evaluated using control charts and standard deviation analysis, while thermal noise, expressed as $V_{n}=\sqrt{4kTBR}$ , has limited accuracy at higher resistances.^29^ Enhancing hydrogel conductivity while maintaining mechanical integrity is therefore essential for stable signal transmission [R7]. Furthermore, recent studies highlight that integrating self-healing, fatigue resistance, and environmental durability into hydrogel sensors can substantially improve their long-term reliability and usability in real-world applications.^30^

Building on our prior hydrogel platform that addressed adhesion in humid skin and antiswelling mechanics via cellulose nanocrystals (CNC)-reinforced PVA/PAA with tree-frog microstructures,^31^ the present work targets the electrical failure modes that limit long-term accuracy. We demonstrate that DMSO-modified PEDOT:PSS (optimal ∼ 5 wt %) reduces resistivity and suppresses signal drift over 21 days, while a biaxial strain-gauge topology aligned to anatomical deformation outperforms rosette layouts during multiplane shoulder motion (errors < ∼6°). This materials-plus-geometry approach converts a mechanically robust patch into a signal-stable wearable suitable for real-time training feedback and rehabilitation.

Recent studies have emphasized the multifunctionality of conductive hydrogels in flexible wearable sensing. Li et al.^32^ reviewed the performance and design strategies of flexible conductive hydrogels for strain and pressure sensors, highlighting the need for structural optimization alongside conductivity enhancement. Wu et al.^33^ reported that future wearable hydrogels must integrate long-term signal stability and deformation adaptability to achieve practical motion tracking. Guo and Ma^34^ further demonstrated that conductive nanocomposite hydrogels can achieve high sensitivity (gauge factors up to ∼15–20 and conductivity above 10^–1^ S cm^–1^) when synergistically designed with flexible geometries.

Unlike previous studies that primarily enhanced conductivity or modified structural designs separately, this work introduces a dual strategy combining chemical modification and sensor topology optimization. Specifically, dimethyl sulfoxide (DMSO) is utilized to modify poly­(3,4-ethylenedioxythiophene):poly­(styrenesulfonate) (PEDOT:PSS), effectively reducing resistivity and suppressing long-term signal drift. Simultaneously, a biaxial strain gauge layout is designed to align with the principal muscle deformation direction, minimizing measurement interference compared to conventional rosette patterns. This integrated approach represents a novel materials-and-design synergy that ensures both high conductivity and stable strain detection for accurate real-time monitoring in wearable applications.

## 2 Experiment and Method

### 2.1 Materials

PVA (MW 27,000–32,000, Emperor Chemical, Taiwan) was the main polymer due to its flexibility and biocompatibility. Acrylic acid (99.5%, Thermo Scientific, United States) and glycerin (Katayama, Japan) acted as cross-linking and plasticizing agents. CNC (CelluForce, Canada) improved reinforcement, while ammonium persulfate (APS, 98%, DAEJUNG, South Korea) and tetramethyl ethylenediamine (TEMED, 99%, Alfa Aesar, United States) initiated polymerization. *N*-Methylenebis­(acrylamide) (MBAA, 99%, Sigma-Aldrich, United States) stabilized the network. PEDOT:PSS (Heraeus, Germany) introduced conductivity, dimethyl sulfoxide (DMSO, 99%, Katayama, Japan) enhanced charge transport, and RTV silicone (Modelcrack Studio, Taiwan) provided encapsulation. Together, these components formed a conductive hydrogel composite for wearable sensors.

### 2.2 Enhancement of Hydrogel Conductivity by DMSO

Table 1 shows the detailed composition of all samples. To prepare the conductive hydrogel, 3.0 g of PVA was dissolved in 20 mL of a glycerin–water solution (1:1 v/v) under continuous stirring at 80 °C for 1 h until a clear viscous solution formed. After cooling to 25 °C, 2 mL of AA, 0.05 g of APS, 10 μL of TEMED, 0.1 wt % of MBAA, and 0.5 wt % of CNC were added sequentially to obtain a homogeneous precursor. Separately, 2 mL of PEDOT:PSS was mixed with DMSO (0, 5, 10, or 15 wt %) and ultrasonicated for 10 min to ensure complete dispersion. The DMSO-modified PEDOT:PSS solution was then combined with the PVA/PAA precursor at 0 °C and stirred for 15 min. The mixture was poured into silicone molds (10 × 10 × 50 mm) and cured at 60 °C for 6 h to form covalent cross-links. Finally, the hydrogels underwent three freeze–thaw cycles (−20 °C for 12 h, followed by thawing at 25 °C for 6 h), producing a dual-network hydrogel with enhanced conductivity and structural stability. Scheme 1 shows the full preparation process of the hydrogel.

**Scheme 1:**
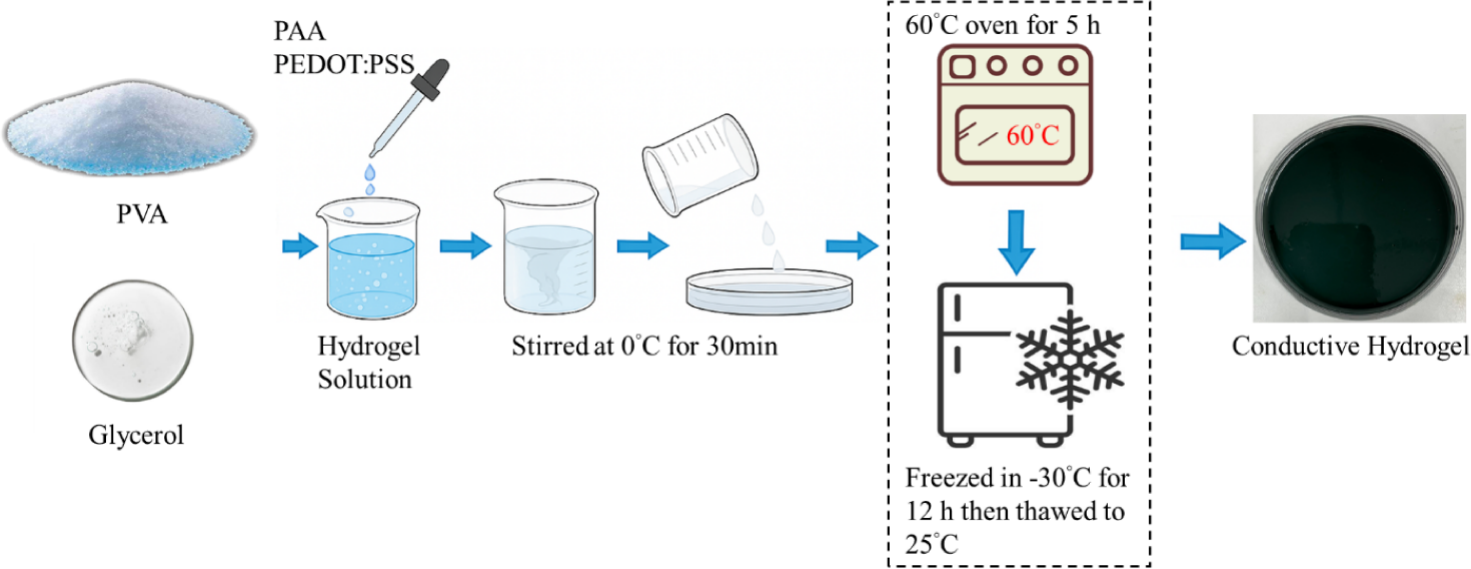
Schematical Diagram of Hydrogel Preparation

**Table 1:** Detailed Composition of the Prepared Hydrogels

Sample code	PVA (g)	CNC (wt %)	PEDOT:PSS (mL)	DMSO (wt %)
DMSO 0	3	0.5	2	0
DMSO 5	3	0.5	2	5
DMSO 10	3	0.5	2	10
DMSO 15	3	0.5	2	15

### 2.3 Improved Strain Gauge Design

Laser cutting produced biaxial and rosette strain gauge circuits on acrylic. Silicone–hardener mixtures cast over the templates, creating molds. PVA/PAA solution was poured into these molds to form flexible sensor patches. This process integrates patterning and casting, enabling the reliable fabrication of wearable strain sensors.

### 2.4 FTIR Analysis

FTIR verified hydrogen bond replacement between PEDOT and PSS by DMSO. Spectra were collected on ZnSe in the 4500–500 cm^–1^ range at 4 cm^–1^ resolution and 16 scans/min. Shifts in characteristic peaks confirmed DMSO substitution.

### 2.5 Conductivity Measurement

Specimens of 10 × 10 × 50 mm were tested with a digital multimeter. Resistance decreased with DMSO addition, confirming enhanced conductivity in the PVA/PAA–PEDOT:PSS hydrogel.

### 2.6 Dynamic Mechanical Analysis (DMA)

DMA measured viscoelasticity and Tg. Storage modulus decreased and tan δ peaked as temperature increased, indicating chain mobility.^26^ Stable networks showed single peaks, while unstable ones showed fluctuations.^35,36^ Specimens (30 × 6 × 0.8 mm) were tested at 1 Hz from 25 to 200 °C at 5 °C/min to evaluate the effect of DMSO.

### 2.7 Human Monitoring Test

MediaPipe detected shoulder motion with 32 landmarks. Angles were expressed in spherical coordinates: θ for the frontal plane and φ for the horizontal plane. Shoulder joint angles from MediaPipe were compared with those from wearable sensors. Arduino Nano signals (0–1023) were calibrated with 0° and 180° as Smin and Smax to convert voltages to angles, enabling real-time monitoring.

### 2.8 Fitness Training Application

During bench press, arm–torso angles above 75° narrowed the subacromial space, causing impingement and rotator cuff strain. Safe motion remained at 45°–75°, maximizing pectoralis activation.^37^ In the shoulder press, lifting arms in the scapular plane (30°–45°) reduced compression and improved deltoid and cuff activation.^38^ Strain rosettes and biaxial gauges monitored these exercises, and correct angles were defined for each motion to compare sensor performance in real time.

### 2.9 Ethical Consideration

This study involved only noninvasive monitoring of human joint motion with minimal risk and no identifiable or medical data collection. According to institutional guidelines, such procedures are exempt from formal ethical review. Written informed consent was obtained from the participant.

## 3 Results and Discussion

### 3.1 Enhancement of Hydrogel Conductivity by DMSO

#### 3.1.1 FTIR Spectral Analysis

FTIR analysis was performed to verify whether DMSO replaced the hydrogen bonds between PEDOT and PSS. The spectra of hydrogels with varying DMSO concentrations were collected in the 1050–1750 cm^–1^ range. Figure 1 shows the FTIR spectra and the DMSO–PEDOT:PSS interaction schematic. As DMSO content increased, the peak at 1150 cm^–1^, attributed to SO_3_^–^ groups of PSS interacting with PEDOT^+^, gradually disappeared. This result indicated that the addition of DMSO disrupted the original PEDOT:PSS bonding structure. The disappearance of this feature demonstrated the weakening of PEDOT–PSS electrostatic interactions.

**Figure 1:**
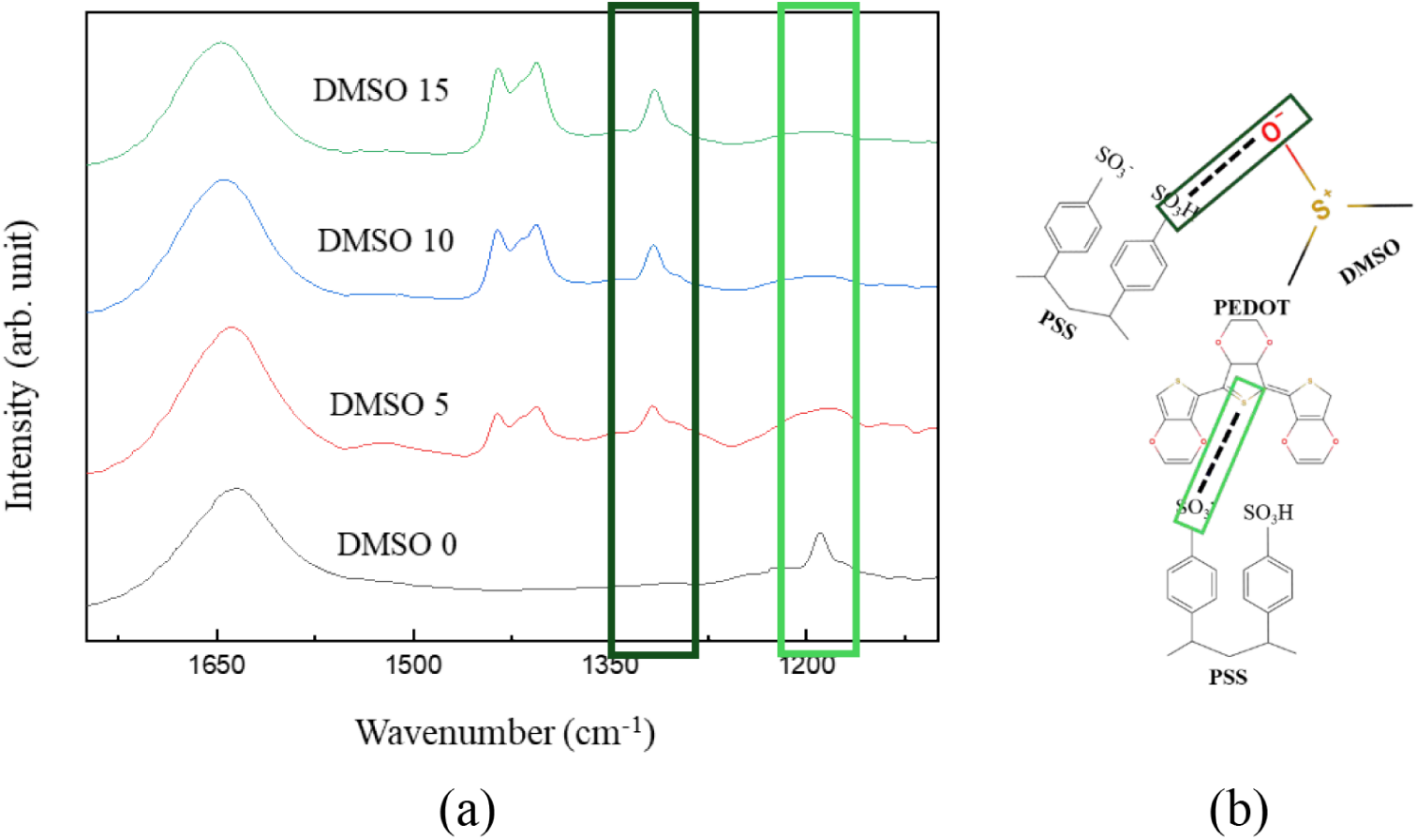
(a) FTIR spectra of hydrogels with different DMSO concentrations in the 1050–1750 cm^–1^ range. (b) Schematic of hydrogen bonding between DMSO and PEDOT:PSS.

Meanwhile, a new absorption peak appeared at 1300 cm^–1^, corresponding to hydrogen bonds between −SO_3_H groups in PSS and oxygen atoms in DMSO. This shift suggests that DMSO established new intermolecular interactions with PSS. Consequently, the modified bonding environment provides enhanced conductive pathways within the hydrogel.

#### 3.1.2 Conductivity Measurement Results

After confirming that DMSO modified the PEDOT:PSS structure, resistance values of hydrogels with different DMSO concentrations were measured using a digital multimeter. Figure 2 presents the conductivity retention and resistivity results. These data allowed the evaluation of how DMSO content influenced electrical properties. As shown in Figure 2, the initial sample without DMSO exhibited a resistance of ∼200 kΩ. With DMSO concentration increasing to 15 wt %, resistance decreased to ∼40 kΩ, approximately 20% of the original value. This reduction demonstrated that conductivity improved significantly with DMSO incorporation.

**Figure 2:**
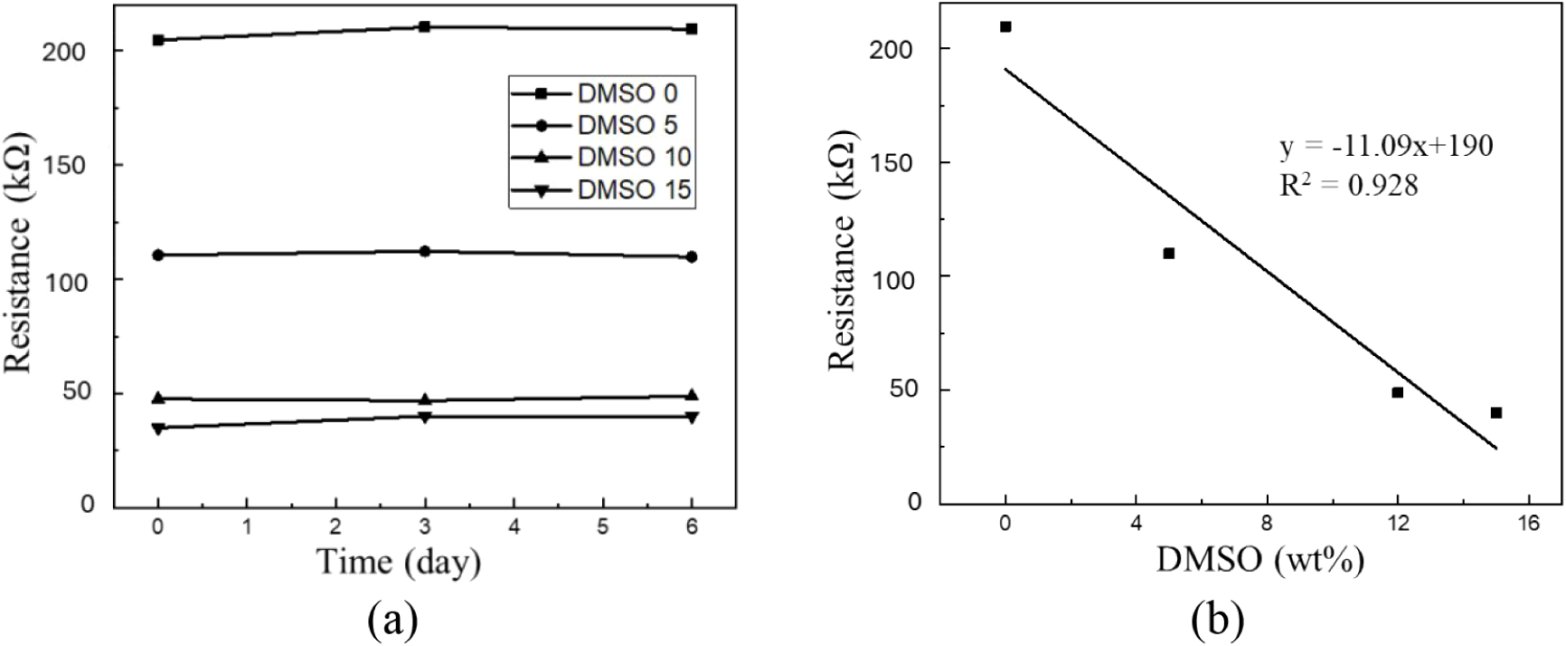
(a) Conductivity retention of hydrogels with different DMSO concentrations. (b) Resistivity measurements as a function of DMSO content.

The mechanism suggests that DMSO disrupted PEDOT–PSS hydrogen bonding, reduced the insulating effect of PSS, and promoted PEDOT chain rearrangement into continuous pathways. Beyond 10 wt %, the resistance decline slowed, implying the replacement reaction’s saturation. Conductivity retention tests over 6 days showed no significant variation, confirming stable performance for wearable sensing applications.

#### 3.1.3 DMA Results

The viscoelastic properties of the PVA/PAA–PEDOT:PSS hydrogels were investigated by DMA to evaluate the influence of DMSO concentration on the mechanical relaxation behavior and network stability (Figure 3). The storage modulus (*G*′) represents the elastic component of the hydrogel, while the loss modulus (*G*″) corresponds to the viscous response. The tan δ (*G*″/*G*′) curve indicates the damping capacity and is often used to determine the Tg of polymeric networks.

**Figure 3:**
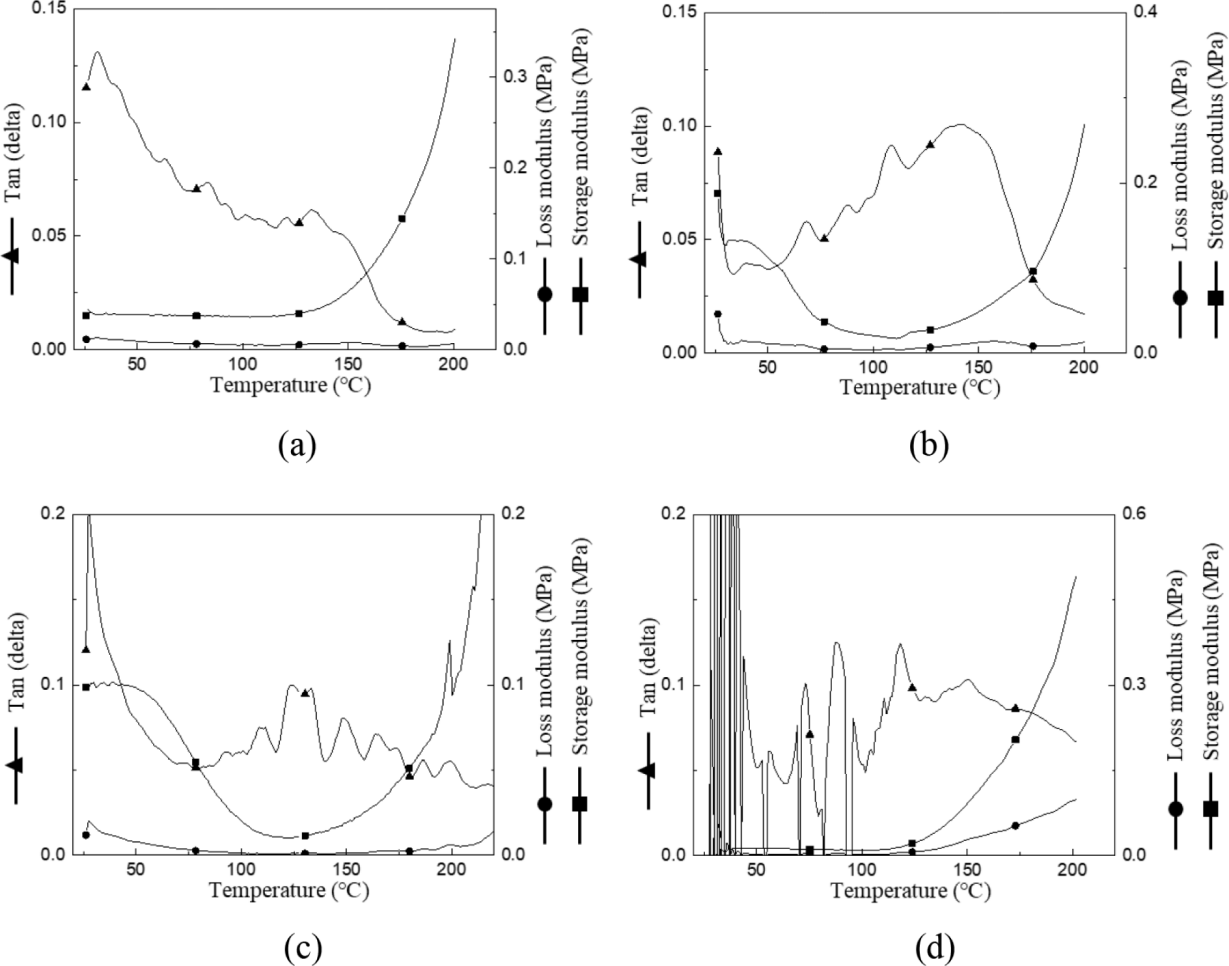
DMA curves of hydrogels with varying DMSO concentrations: (a) 0%, (b) 5%, (c) 10%, (d) 15%.

As the temperature increased, the storage modulus gradually decreased, indicating enhanced chain mobility and the softening of the hydrogel matrix. The tan δ peak appeared between approximately 100 and 150 °C, depending on the DMSO content, marking the transition from the glassy to the rubbery state. Samples with moderate DMSO addition showed a single, well-defined tan δ peak and smooth modulus transition, suggesting a homogeneous polymer network with strong segmental interactions. In contrast, hydrogels containing excessive DMSO exhibited multiple or fluctuating tan δ peaks, reflecting phase separation or microstructural instability likely caused by overplasticization of the polymer chains.

The incorporation of DMSO played a dual role: it enhanced the conductivity of PEDOT:PSS by improving chain alignment and ion mobility, yet at high concentrations it disrupted hydrogen bonding between PVA and PAA, weakening the mechanical integrity. This is consistent with the observed decrease in G′ and the broadening of tan δ peaks at higher DMSO contents. Such results confirm that an optimal DMSO concentration is essential to balance electrical enhancement and structural stability.

### 3.2 Improved Strain Gauge Design

#### 3.2.1 Preparation of Sensor Patches

CO_2_ laser cutting was used to fabricate sensor designs and circuits on 3 mm thick transparent acrylic plates. Two models, a biaxial strain gauge and a strain rosette, were produced for subsequent measurements. Figure 4 shows both the acrylic models and the silicone molds cast from them. The acrylic models were filled with silicone mixtures, cured, and demolded to form precise casting molds. These molds provided cavities for fabricating hydrogel-based strain sensor patches. The process ensured reproducibility and compatibility with multiple design configurations. The final patches are shown in Figure 5. The transparent regions represented the nonconductive PVA/PAA hydrogel body, while the dark areas contained PEDOT:PSS solution that connected to external circuits. These conductive zones functioned as the sensing elements for strain detection.

**Figure 4:**
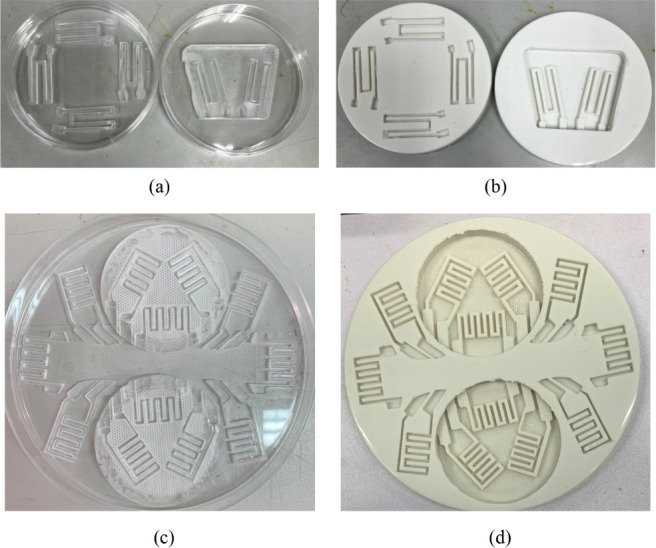
(a) Acrylic model of a biaxial strain gauge. (b) Silicone mold of a biaxial strain gauge. (c) Acrylic model of a strain rosette. (d) Silicone mold of a strain rosette.

**Figure 5:**
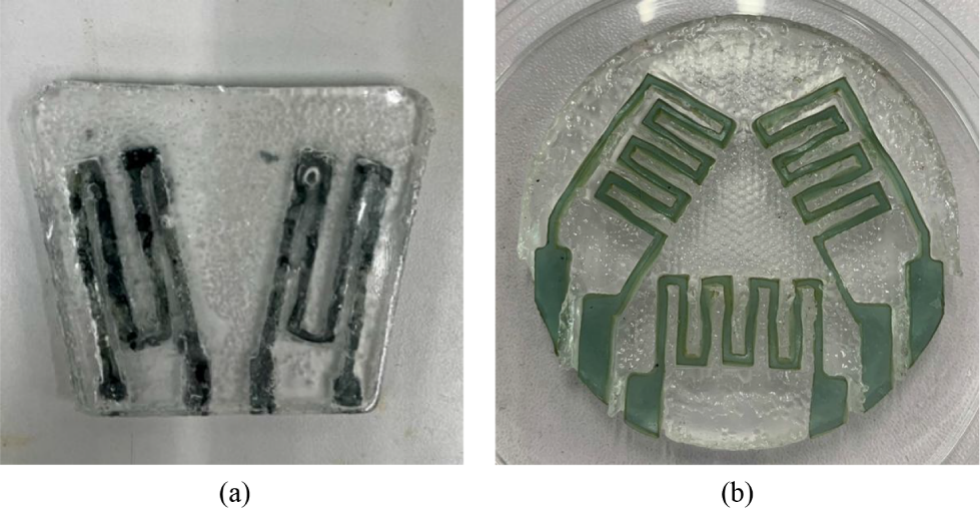
(a) Completed biaxial strain gauge patch. (b) Completed strain rosette patch.

#### 3.2.2 Human Real-Time Monitoring System

Sensor patches were attached to the shoulder joint to measure dynamic motion in different planes. The strain rosette and the biaxial strain gauge were tested, and their results were compared with video-derived joint angles. Figures 6 and 7 present dynamic tracking curves for the frontal and horizontal planes. In the frontal plane (Figure 6), both sensors showed stable tracking with average errors of 5.9° for the strain rosette and 5.7° for the biaxial gauge. In the horizontal plane (Figure 7), errors were 4.9° and 4.6°, respectively, matching the camera reference well. These results indicated that both sensors achieved accurate detection during single-plane shoulder motions.

**Figure 6:**
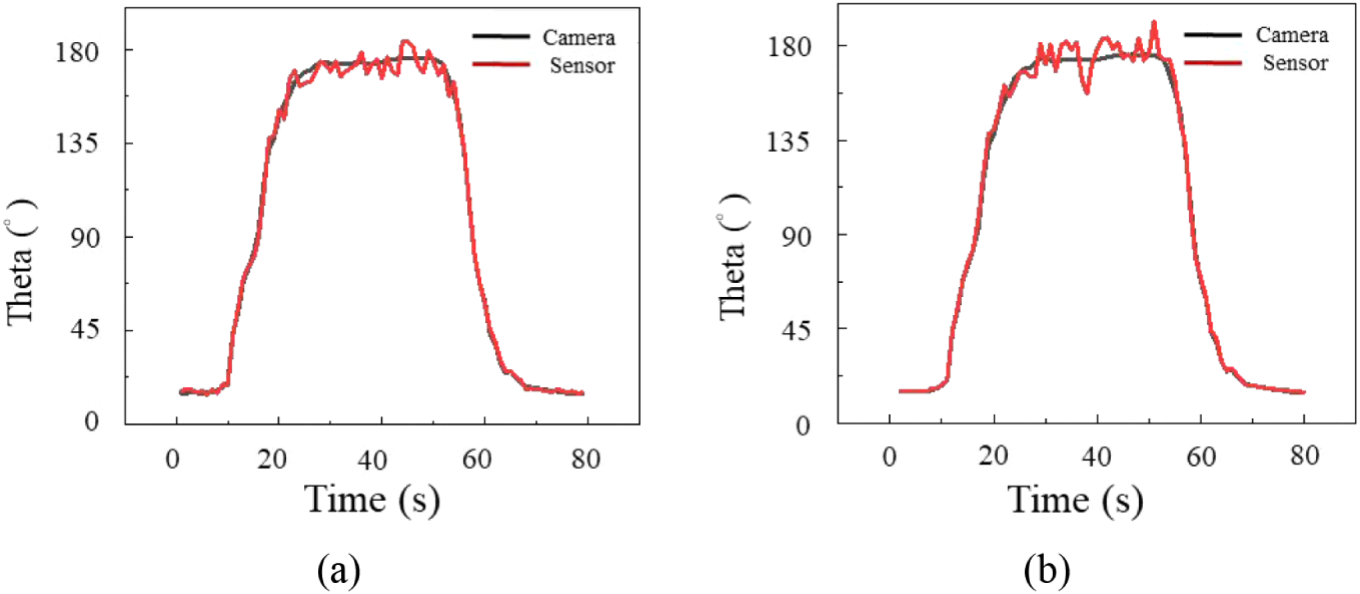
Frontal plane motion measured by (a) strain rosette and (b) biaxial strain gauge.

**Figure 7:**
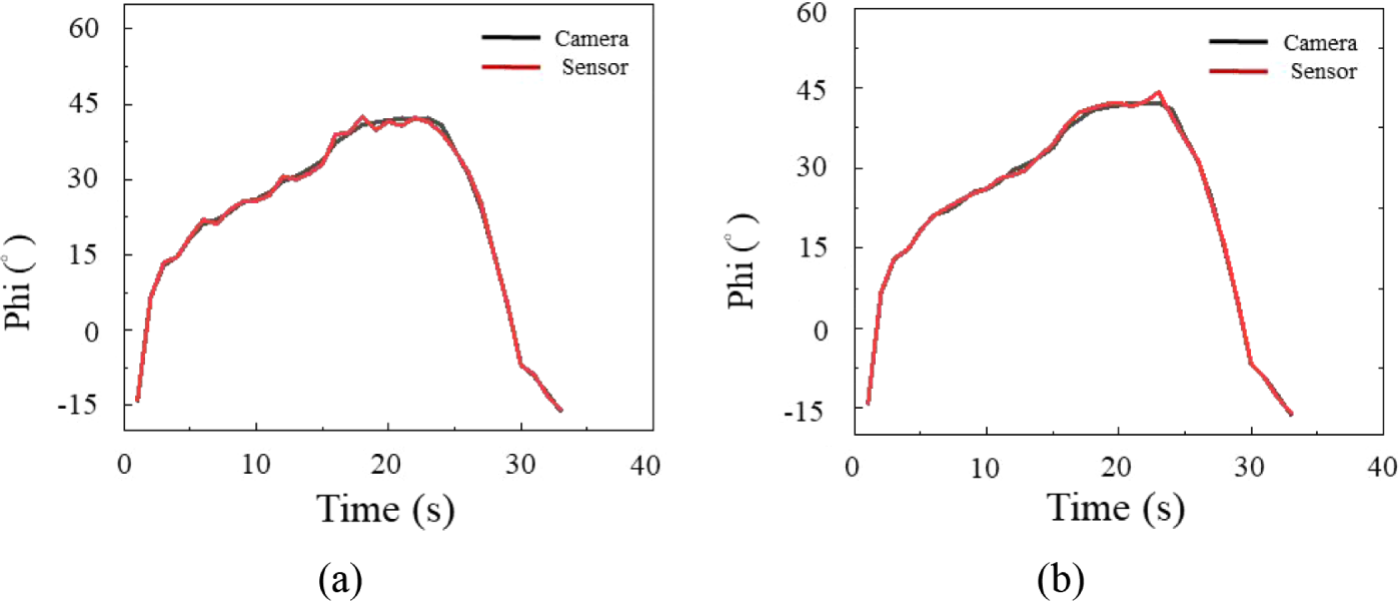
Horizontal plane motion measured by (a) strain rosette and (b) biaxial strain gauge.

A shoulder circumduction test was performed by tracing circles of different sizes in three trials to assess complex motion further. Figures 8 and 9 show polar and azimuthal angle variations compared with camera data. Errors increased under multiplane deformation, confirming that complex strain states challenged measurement accuracy. For the polar angle, average errors were 8.3° for the rosette and 5.8° for the biaxial gauge, while for the azimuthal angle, errors were 7.8° and 6.1°. The biaxial gauge achieved higher accuracy, with deviations below 6° in both dimensions. This phenomenon supported the hypothesis that alignment with muscle deformation direction improved measurement fidelity.

**Figure 8:**
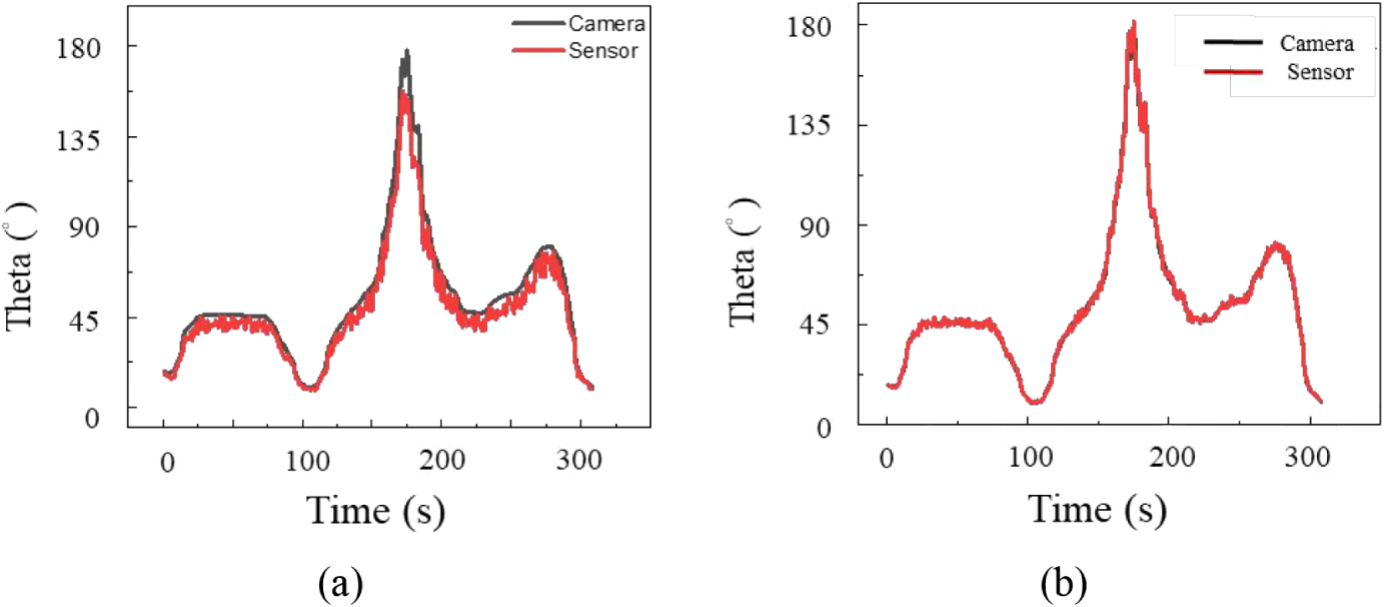
Polar angle variation during circumduction using (a) a strain rosette and (b) a biaxial strain gauge.

**Figure 9:**
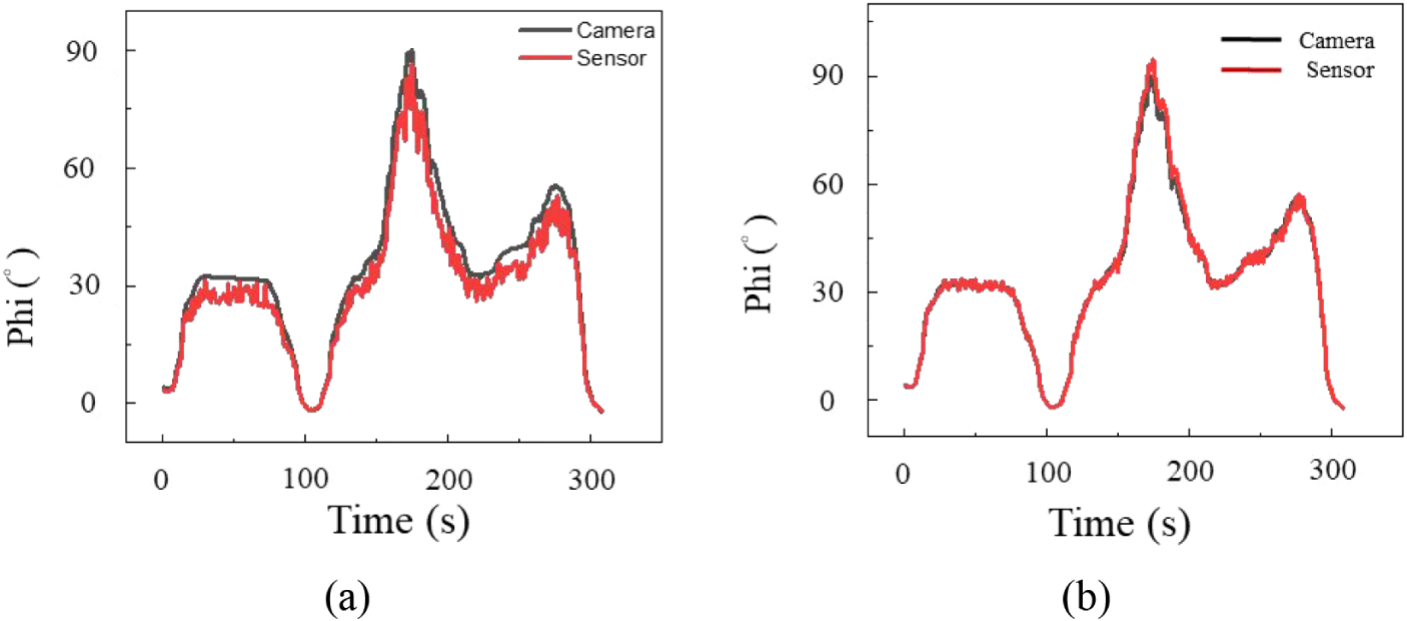
Azimuthal angle variation during circumduction using (a) a strain rosette and (b) a biaxial strain gauge.

The normal strain measured by a sensing element oriented at an angle θ relative to the reference axis can be described using the strain transformation relationship, as follows:

1 $$\varepsilon_{\theta }=\varepsilon_{x}\cos^{2}\theta +\varepsilon_{y}\sin^{2}\theta +\frac{\gamma_{\mathrm{xy}}}{2}\sin(2\theta )$$

where ε_θ_ denotes the normal strain measured along the sensing direction, ε*_x_* and ε*_y_* represent the normal strain components along the reference and orthogonal in-plane directions, respectively, γ*_xy_* is the in-plane shear strain, and θ is the orientation angle between the sensing element and the reference axis.

The mechanism suggests that the strain rosette, though capable of detecting multidirectional strains, introduced cross-interference from shear and secondary strains on curved surfaces. According to eq 1, when θ ≠ 0°, the principal strain measurement combines ε_*x*_, ε_γ_, and γ_*x*γ_, causing deviations from actual contraction. In contrast, the biaxial gauge, with a simpler orientation-specific design aligned to muscle fibers, reduced interference and produced more reliable strain readings. Table 2 summarizes representative conductive hydrogels recently reported for wearable sensing. The comparison includes conductivity, sensitivity, stability, and stretchability. Although the present DMSO-modified PEDOT:PSS hydrogel shows a moderate conductivity of 0.125 mS·cm^–1^, it achieves long-term stability (21 days) and low angular error (<6°) during multiplane motion, outperforming many reported systems in terms of signal fidelity and practical usability. This balanced performance indicates that optimizing both material conductivity and sensor geometry can yield a reliable, soft, and durable platform suitable for real-time human-motion monitoring.

**Table 2:** Benchmarking Conductive Hydrogels for Wearable Sensing

Study (Year)	Conductive Component	Electrical Performance	Sensing Performance	Stability (cycles/days)	Notes
**This work**	DMSO-modified PEDOT:PSS in PVA/PAA	0.125 mS·cm^–1^	Angle error <6° (multiplane shoulder)	21 days (control charts within ±2σ)	Materials + biaxial gauge design; real-time Motion fidelity
Lu et al., (2019)^39^	Pure PEDOT:PSS (DMSO-assisted nanofibrils)	∼20 S·cm^–1^ (PBS), ∼ 40 S·cm^–1^ (DI water)	-	Stable over months in aqueous media	High intrinsic conductivity of pristine conducting polymer hydrogels
Huo et al., (2024)^40^	PAA/CS-DOPA–Zn^2+^	0.88 S·m^–1^	GF = 1.14 (<300%), 2.92 (300–700%), 7.34 (700–850%), 25.18 (850–970%)	-	Strong self-adhesion + wide strain range
Wu et al., (2024)^41^	MXene-based PVA/PAA hydrogel	∼25 mS·cm^–1^	GF ≈ 4.75, response ∼ 360 ms	-	Typical MXene balance of conductivity and sensitivity
Shinde et al. (2025)^42^	Tough conductive hydrogel	-	GF ≈ 1.67 (linear, *R* ^2^ ≈0.97)	10,000 cycles (stable)	Low hysteresis under repeated loading
Wu et al., 2025)^33^	Doped conductive ionic hydrogels	∼1.8 × 10^–5^ S·cm^–1^	-	-	Shows lower conductivity end of hydrogel spectrum

### 3.3 Stability Measurement of Conductivity-Enhanced Sensors

Stability was defined as the ability of the sensor to maintain its initial accuracy after long-term operation. Accuracy was evaluated by comparing each measurement with the camera reference, calculating mean errors and standard deviations as indicators. Control charts with upper and lower control limits (UCL and LCL) were drawn to examine whether data remained within acceptable ranges. For testing, four shoulder angles (45°, 90°, 135°, 180°) were measured, each repeated 30 times. To assess long-term stability, the same tests were repeated on day 7, day 14, and day 21. Sensors without DMSO and with 5 wt % DMSO were compared to determine differences.

Figure 10 shows that sensors without DMSO displayed apparent degradation over time. By day 7, error curves at 45° and 90° exceeded control limits, while all angles showed unstable outputs by days 14 and 21. The mechanism suggests that water loss and network deterioration reduced conductivity, producing inconsistent resistance and accuracy. Figure 11 shows the results for the 5 wt % DMSO sensor. Most errors remained within ±2σ*E* over 21 days, with only minor deviations at 90° on day 21. The DMSO-enhanced sensor maintained higher stability and reproducibility than the untreated sample. These findings confirmed that DMSO improved the conductive network, reduced resistance fluctuation, and minimized signal drift. The enhanced hydrogel provided reliable performance during extended use, supporting the hypothesis that conductivity reinforcement directly strengthened long-term stability. Thus, DMSO doping offered an effective strategy to ensure durability for wearable sensing applications.

**Figure 10:**
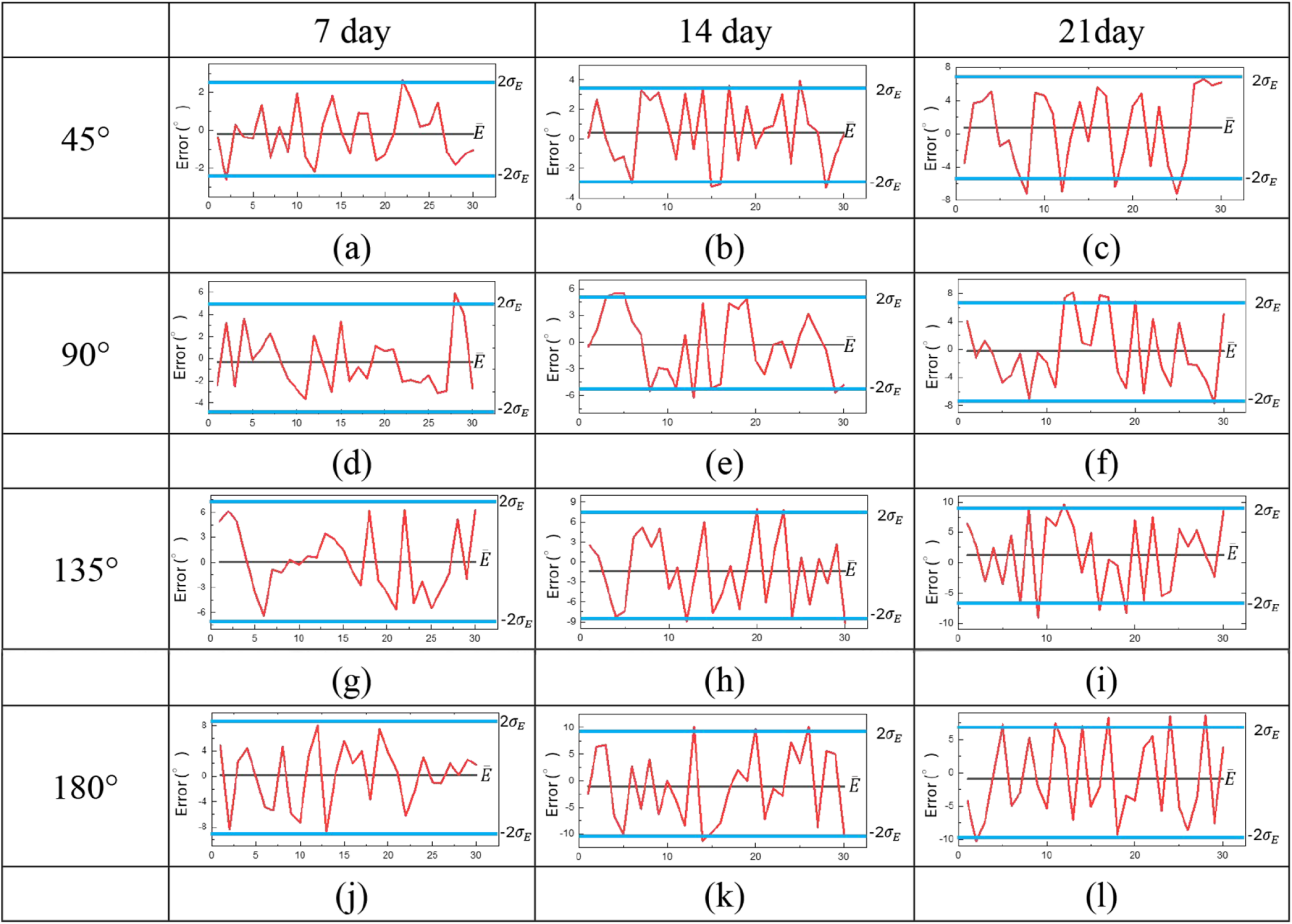
Stability of hydrogel sensor without DMSO at 45°, 90°, 135°, and 180° on days 7, 14, and 21.

**Figure 11:**
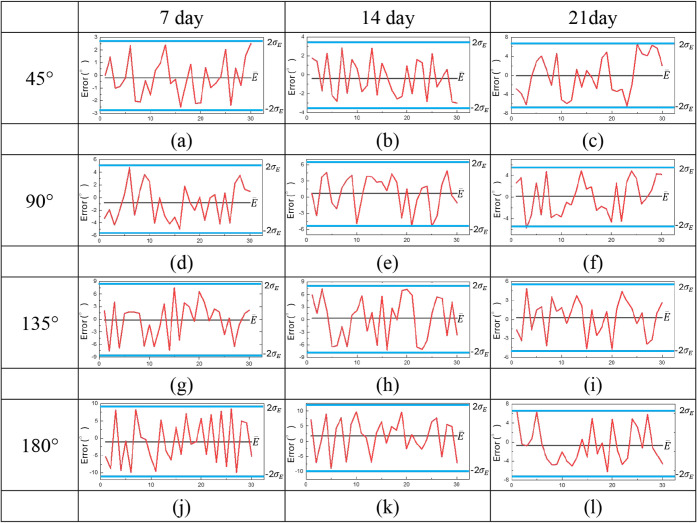
Stability of hydrogel sensor with 5 wt % DMSO at 45°, 90°, 135°, and 180° on days 7, 14, and 21.

### 3.4 Fitness Training Application—Bench Press and Shoulder Press

This study applied hydrogel sensors to bench press and shoulder press exercises. Flexible patches were placed near the shoulder joint, and signals were transmitted via Arduino and Bluetooth to a mobile application for real-time monitoring. The app displayed joint angles, provided motion feedback, and issued warnings when abnormal postures occurred.

Figure 12 shows the measurement results during the bench press. Safe movement requires the arm–torso angle to remain between 45° and 75°, since exceeding 75° narrows the subacromial space and risks impingement. Within the recommended range, shoulder compression was reduced and pectoralis activation was maximized. During the shoulder press, safe posture also required specific arm orientation (Figure 13). Arms should be elevated in the scapular plane at 30°–45° rather than parallel to the torso, which minimizes compression and improves deltoid activation. The application used these thresholds to trigger red warnings when angles exceeded limits and green signals when posture was correct.

**Figure 12:**
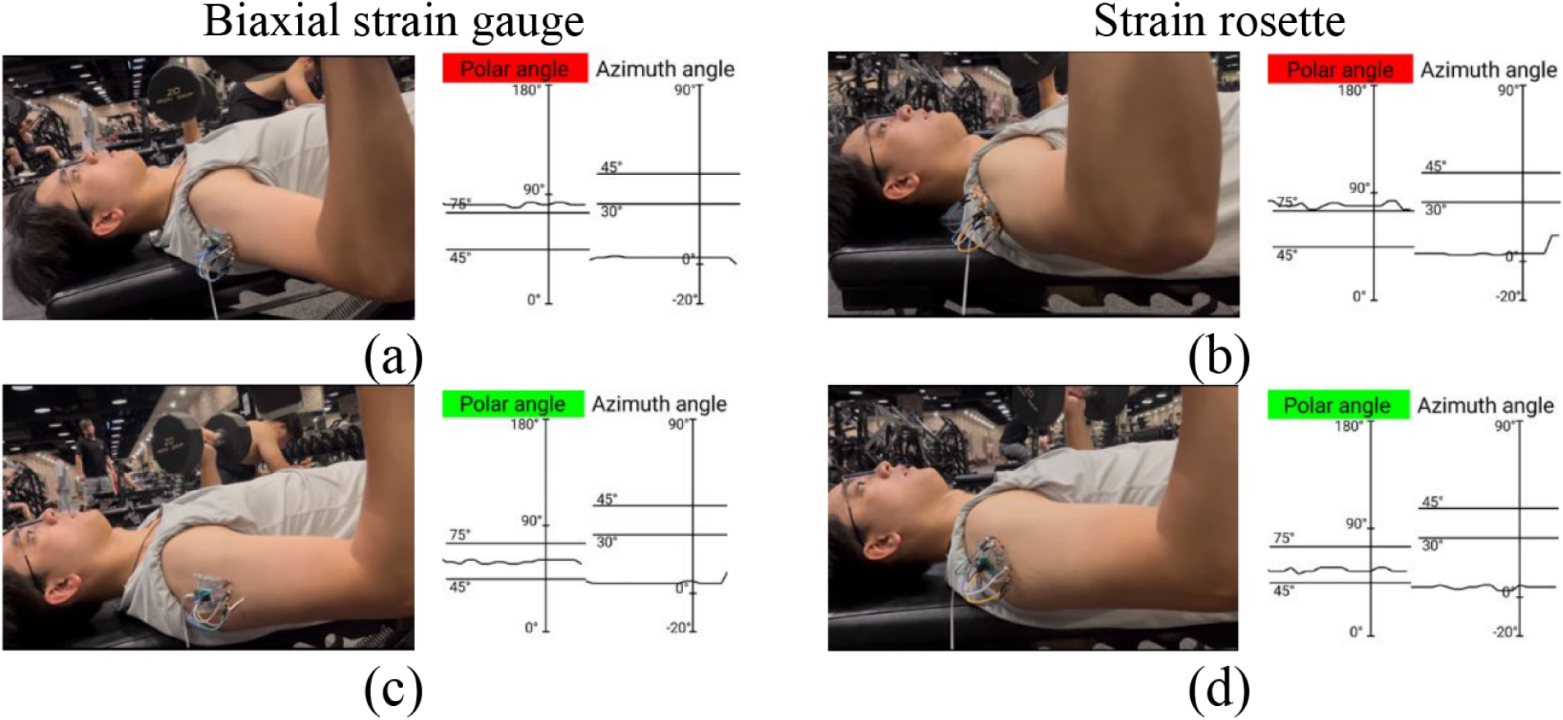
Bench press monitoring with (a) biaxial strain gauge >75°, (c) biaxial strain gauge 45°–75°, (b) strain rosette >75°, and (d) strain rosette 45°–75°.

**Figure 13:**
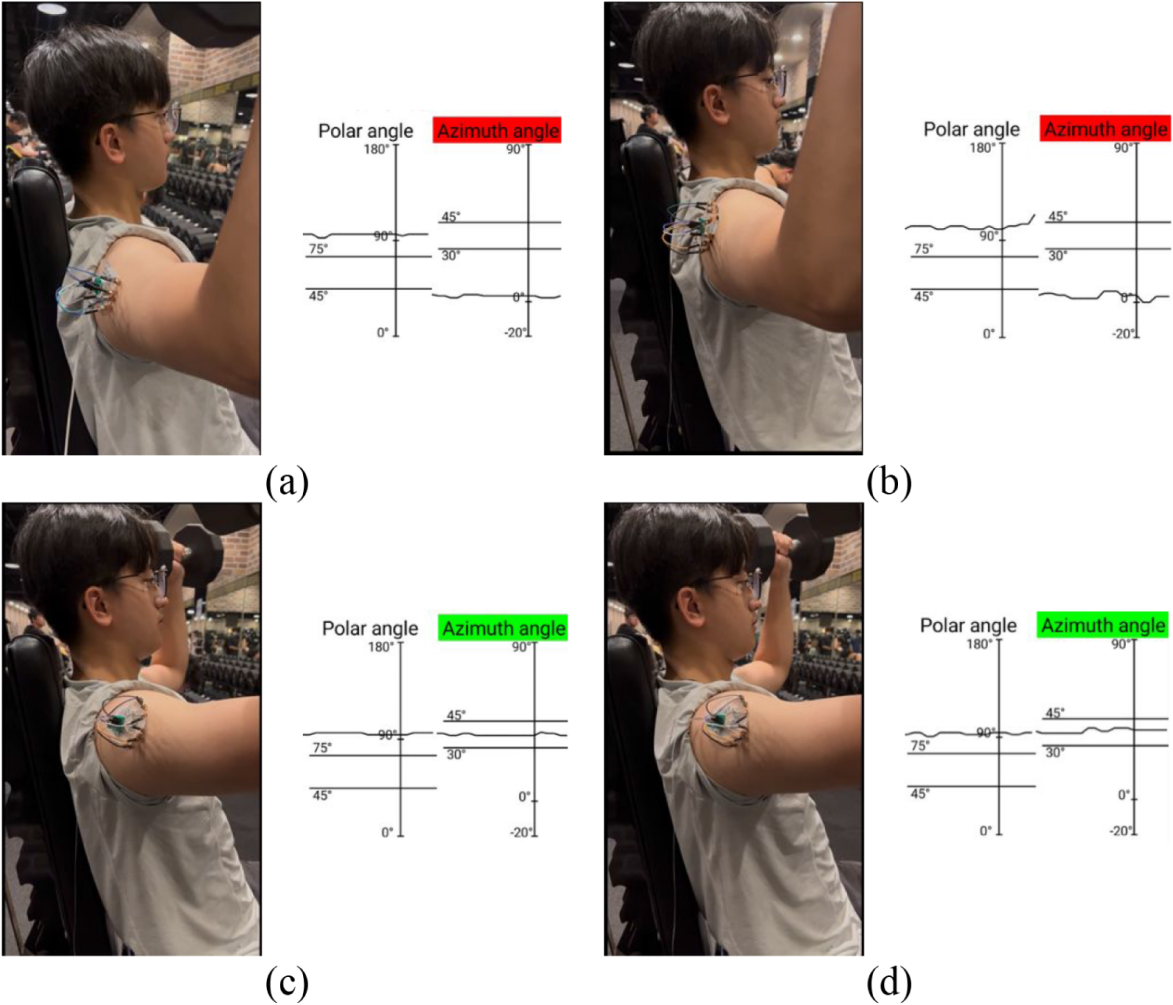
Shoulder press monitoring with (a) biaxial strain gauge <30°, (c) biaxial strain gauge 30°–45°, (b) strain rosette <30°, and (d) strain rosette 30°–45°. Photograph courtesy of Yen-Kai Huang. Copyright 2025.

The biaxial strain gauge provided smoother feedback curves from the measurements than the strain rosette. The rosette was more sensitive to interference and less accurate under dynamic motion, which amplified errors in real-time monitoring. These findings indicated that the biaxial design offered greater reliability for training feedback and injury prevention.

## 4 Conclusions

This study hypothesized that improving hydrogel conductivity with DMSO and optimizing strain gauge design would enhance the accuracy and stability of wearable motion sensors. FTIR analysis showed that DMSO replaced hydrogen bonds between PEDOT and PSS, while resistivity tests confirmed a reduction of up to 80% at 15 wt %. DMA results demonstrated that 5 wt % DMSO provided the best conductivity and structural stability balance, whereas higher concentrations disrupted the PVA/PAA network. It was further hypothesized that aligning strain gauge orientation with muscle deformation would reduce angular error. Experiments indicated that in single-plane motions, both biaxial and rosette gauges achieved mean errors below 6°. In multiplane circumduction, the biaxial gauge performed better, with errors of 5.8° and 6.1° in polar and azimuthal angles, compared with 8.3° and 7.8° for the rosette, confirming the importance of directional design.

The final hypothesis proposed that conductivity reinforcement would improve long-term stability. Control chart analysis revealed that untreated hydrogels degraded after 14 days, while 5 wt % DMSO samples maintained consistent performance for 21 days within ±2σ. This work demonstrates that combining conductivity enhancement with biaxial strain gauge design enables accurate, stable, and practical wearable sensors. Future studies will refine materials and extend applications to rehabilitation and personalized training.

## References

[1] Corzo D., Tostado-Blázquez G., Baran D. (2020). Flexible Electronics:
Status, Challenges and Opportunities. Front.
Electron..

[2] Banitaba S. N., Khademolqorani S., Jadhav V. V., Chamanehpour E., Mishra Y. K., Mostafavi E., Kaushik A. (2023). Recent
progress of bio-based smart wearable sensors for healthcare applications. Mater. Today Electron..

[3] Liu Y., Wang L., Mi Y., Zhao S., Qi S., Sun M. (2022). Transparent
stretchable hydrogel sensors: materials,
design and applications. J. Mater. Chem. C.

[4] Ding H., Liu J., Shen X., Li H. (2023). Advances in the Preparation of Tough
Conductive Hydrogels for Flexible Sensors. Polymers.

[5] Rahmadiawan D., Shi S.-C., Zhuang W.-T. (2024). Reinforcing polyvinyl alcohol films
with layered double hydroxide and tannic acid to enhance tensile strength,
tribological performance, and corrosion resistance in biomedical coating
applications. Mater. Res. Express.

[6] Li G., Li C., Li G., Yu D., Song Z., Wang H., Liu X., Liu H., Liu W. (2022). Development of Conductive
Hydrogels for Fabricating Flexible Strain Sensors. Small.

[7] Kang L.-L., Xue M., Liu Y.-Y., Yu Y.-H., Liu Y.-R., Li G. (2022). Proton conductive
metal–organic frameworks based on main-group metals. Coord. Chem. Rev..

[8] Gasni D., Rahmadiawan D., Irwansyah R., Khalid A. E. (2024). Composite of Carboxymethyl
Cellulose/MXene and Span 60 as Additives to Enhance Tribological Properties
of Bio-Lubricants. Lubricants.

[9] Rahmadiawan D., Abral H., Kotodeli R. A., Sugiarti E., Muslimin A. N., Admi R. I. (2023). A Novel
Highly Conductive, Transparent, and Strong
Pure-Cellulose Film from TEMPO-Oxidized Bacterial Cellulose by Increasing
Sonication Power. Polymers.

[10] Wu Y., Sun S., Geng A., Wang L., Song C., Xu L., Jia C., Shi J., Gan L. (2020). Using TEMPO-oxidized-nanocellulose
stabilized carbon nanotubes to make pigskin hydrogel conductive as
flexible sensor and supercapacitor electrode: Inspired from a Chinese
cuisine. Compos. Sci. Technol..

[11] Li T., Liang B., Ye Z., Zhang L., Xu S., Tu T. (2022). An integrated
and conductive hydrogel-paper patch for
simultaneous sensing of Chemical-Electrophysiological signals. Biosens. Bioelectron..

[12] Fauza A. N., Qalbina F., Nurdin H., Ambiyar A., Refdinal R. (2023). The influence
of processing temperature on the mechanical properties of recycled
PET fibers. Teknomekanik.

[13] Martin A., Taer E., Nasruddin N., Khotimah N. (2025). High-pressure adsorption
isothermal on a novel microporous material from polyethylene terephthalate
plastic waste in carbon dioxide capture applications. Teknomekanik.

[14] Salim O., Mahmoud K. A., Pant K. K., Joshi R. K. (2019). Introduction to
MXenes: synthesis and characteristics. Mater.
Today Chem..

[15] Rahmadiawan D., Abral H., Nasruddin N., Fuadi Z. (2021). Stability, Viscosity,
and Tribology Properties of Polyol Ester Oil-Based Biolubricant Filled
with TEMPO-Oxidized Bacterial Cellulose Nanofiber. Int. J. Polym. Sci..

[16] Shi H., Liu C., Jiang Q., Xu J. (2015). Effective Approaches to Improve the
Electrical Conductivity of PEDOT: PSS: A Review. Adv. Electron. Mater..

[17] Shahrim N. A. A., Ahmad Z., Wong Azman A., Fachmi Buys Y., Sarifuddin N. (2021). Mechanisms for doped PEDOT: PSS electrical
conductivity
improvement. Mater. Adv..

[18] Yu Z., Xia Y., Du D., Ouyang J. (2016). PEDOT: PSS Films with Metallic Conductivity
through a Treatment with Common Organic Solutions of Organic Salts
and Their Application as a Transparent Electrode of Polymer Solar
Cells. ACS Appl. Mater. Interfaces.

[19] Kim Y. H., Sachse C., Machala M. L., May C., Müller-Meskamp L., Leo K. (2011). Highly Conductive PEDOT:
PSS Electrode with Optimized Solvent and
Thermal Post-Treatment for ITO-Free Organic Solar Cells. Adv. Funct. Mater..

[20] Cruz-Cruz I., Reyes-Reyes M., Aguilar-Frutis M. A., Rodriguez A. G., López-Sandoval R. (2010). Study of the effect of DMSO concentration
on the thickness of the PSS insulating barrier in PEDOT: PSS thin
films. Synth. Met..

[21] Mi Y., Tong W., Lu Y., Cao X., Wang N. (2024). Robust conductive
hydrogel advances self-powered intelligent sports monitoring and fair
judging. Chem. Eng. J..

[22] Al-Dahiree O. S., Tokhi M. O., Hadi N. H., Hmoad N. R., Ghazilla R. A. R., Yap H. J., Albaadani E. A. (2022). Design and Shape Optimization of
Strain Gauge Load Cell for Axial Force Measurement for Test Benches. Sensors.

[23] Ajovalasit A. (2011). Advances in
Strain Gauge Measurement on Composite Materials. Strain.

[24] Yang Y., Wang H., Hou Y., Nan S., Di Y., Dai Y., Li F., Zhang J. (2022). MWCNTs/PDMS composite
enabled printed flexible omnidirectional strain sensors for wearable
electronics. Compos. Sci. Technol..

[25] Zullo G., Silvestroni A. L., Candiotto G., Koptyug A., Petrone N. (2021). A Novel Multi-Axial
Pressure Sensor Probe for Measuring Triaxial Stress States Inside
Soft Materials. Sensors.

[26] Li M., Pu J., Cao Q., Zhao W., Gao Y., Meng T. (2024). Recent advances in hydrogel-based flexible strain sensors for harsh
environment applications. Chem. Sci..

[27] Kim D. H., Ghaffari R., Lu N., Rogers J. A. (2012). Flexible and stretchable
electronics for biointegrated devices. Annu.
Rev. Biomed. Eng..

[28] Someya T., Bao Z., Malliaras G. G. (2016). The rise
of plastic bioelectronics. Nature.

[29] Lall, P. ; Goyal, K. ; Narangaparambil, J. Accuracy, Hysteresis and Extended Time Stability of Additively Printed Temperature and Humidity Sensors. In 2020 IEEE 70th Electronic Components And Technology Conference (ECTC); IEEE, 2020, pp. 1070–1080. DOI: 10.1109/ECTC32862.2020.00173.

[30] Prameswati A., Nurmaulia Entifar S. A., Han J. W., Wibowo A. F., Kim J. H., Sembiring Y. S. B., Park J., Lee J., Lee A.-Y., Song M. H. (2023). Self-Healable Conductive Hydrogels with High Stretchability
and Ultralow Hysteresis for Soft Electronics. ACS Appl. Mater. Interfaces.

[31] Shi S.-C., Cheng S.-T., Rahmadiawan D. (2024). Developing biomimetic PVA/PAA hydrogels
with cellulose nanocrystals inspired by tree frog structures for superior
wearable sensor functionality. Sens. Actuators,
A.

[32] Li Q., Tian B., Liang J., Wu W. (2023). Functional conductive
hydrogels: from performance to flexible sensor applications. Mater. Chem. Front..

[33] Wu J., Hong J., Gao X., Wang Y., Wang W., Zhang H. (2025). Recent Progress in Flexible Wearable Sensors Utilizing
Conductive Hydrogels for Sports Applications: Characteristics, Mechanisms,
and Modification Strategies. Gels.

[34] Guo W.-Y., Ma M.-G. (2024). Conductive nanocomposite
hydrogels for flexible wearable sensors. J.
Mater. Chem. A.

[35] Wang T., Chen H., Liang W. J., Ng B. S. L., Lu R., Qi J., Wang H., Zhang J., Xie H., Xiao R. (2024). Layered Composites for High Tan Delta Plateau over Wide Temperature
Range. Polymers.

[36] <dma-5.pdf/>.

[37] Noteboom L., Belli I., Hoozemans M. J. M., Seth A., Veeger H. E. J., Van Der Helm F. C. T. (2024). Effects of bench press technique
variations on musculoskeletal shoulder loads and potential injury
risk. Front. Physiol..

[38] Blazkiewicz M., Hadamus A. (2022). The Effect of the Weight and Type of Equipment on Shoulder
and Back Muscle Activity in Surface Electromyography during the Overhead
Press-Preliminary Report. Sensors.

[39] Lu B., Yuk H., Lin S., Jian N., Qu K., Xu J., Zhao X. (2019). Pure PEDOT: PSS hydrogels. Nat.
Commun..

[40] Huo H., Shi H., Yang H., Zhang X., Wan J., Shen J. (2024). A conductive
hydrogel with excellent self-adhesion, sensitivity,
and stability for wearable strain sensors to monitor human motion. J. Mater. Chem. A.

[41] Wu W., Zeng Y. P., Tian B., Liang J. (2024). MXene-Based Dual Network
Hydrogel as Flexible Strain Sensor for Human Actions Recognition. IEEE J. Flexible Electro..

[42] Shinde S., Lee H. E. (2025). Wearable Strain
Sensors via Tough and Conductive Hydrogel-Based
MoS2 Composites for Real-Time Motion Tracking. ACS Omega.

